# Emotion Regulation and the Experience of Future Negative Mood: The Importance of Assessing Social Support

**DOI:** 10.3389/fpsyg.2018.02287

**Published:** 2018-11-20

**Authors:** Tracy C. d’Arbeloff, Katherine R. Freedy, Annchen R. Knodt, Spenser R. Radtke, Bartholomew D. Brigidi, Ahmad R. Hariri

**Affiliations:** Laboratory of NeuroGenetics, Department of Psychology & Neuroscience, Duke University, Durham, NC, United States

**Keywords:** cognitive reappraisal, expressive suppression, social support, depression, anxiety

## Abstract

Emotion regulation refers to the use of various strategies, such as cognitive reappraisal and expressive suppression, to help manage our negative experiences, emotions, and thoughts. Although such emotion regulation often occurs within broader social dynamics and interactions, little is known about how social contexts interact with specific regulation strategies to shape the experience of negative emotions. Using data from 544 young adult university students, we provide initial evidence that habitual use of cognitive reappraisal is associated with lower future experience of depression and anxiety primarily through higher perceived social support (PSS). In contrast, expressive suppression is associated with higher future depression and anxiety primarily through lower PSS. These patterns are consistent with the importance of interpersonal influences on emotion regulation and suggest that assessment of social support can help elucidate the mechanisms of successfully regulating negative mood.

## Introduction

Emotion regulation generally refers to the use of cognitive strategies to help manage our negative experiences, emotions, and thoughts (Gross and John, 2003). Two common forms of emotion regulation are cognitive reappraisal and expressive suppression. The first form, cognitive reappraisal, centers around cognitively engaging with and reframing potentially aversive events to mitigate their negative emotional impact (Gross and John, 2003; Marroquín and Nolen-Hoeksema, 2015). Those who habitually use reappraisal tend to have lower symptoms of depression and anxiety, higher self-esteem and overall well-being, better interpersonal relationships, and better coping skills (Gross and John, 2003; Joormann and Gotlib, 2010; Cutuli, 2014). The second common form of emotion regulation, expressive suppression, relies on actively suppressing, or inhibiting emotional expressivity, so as to appear unaffected (Gross and John, 2003). In contrast to cognitive reappraisal, those who habitually use suppression tend to have increased symptoms of depression and anxiety, poor social support networks or fewer close relationships, higher avoidance tendencies, and lower self-esteem and life satisfaction (Gross and John, 2003; Joormann and Gotlib, 2010; Berking et al., 2014; Cutuli, 2014; Sloan et al., 2017).

Despite these differences, both reappraisal and suppression are considered intra-personal strategies occurring internally and separate from external influences. Other emotion regulation strategies can depend almost entirely on external, especially social, factors. One example of this is inter-personal emotion regulation (IER), where an individual attempts to attenuate their emotional experiences through empathetic, pro-social, and supportive social interactions (Zaki and Williams, 2013). Such processes have led to the proposal that intra- and inter-personal regulation strategies exist along a continuum, with some—like reappraisal and suppression—falling closer to the intra-personal end, and others—like IER—closer to the inter-personal end (Zaki and Williams, 2013).

Recent research has recognized that social context may more broadly impact emotion regulation, such that the distinction between intra- and inter-personal strategies are difficult to fully disentangle (Cohen, 2004; Haber et al., 2007; Zaki and Williams, 2013; Marroquín and Nolen-Hoeksema, 2015). The modulatory effects of social context can be active such as that represented by IER or comparably passive such as a person’s awareness of their social networks, sometimes called perceived social support (PSS) (Haber et al., 2007). PSS refers to an individual’s perceptions of the general availability and quality of the social support available to them. As this is a subjective measure, it is often influenced by a person’s perceptions, memories, and judgements, and may only weakly correlate with objective measures of social support (Haber et al., 2007). Perceptions of high social support have been associated with decreased stress, decreased symptoms of depression, increased coping abilities, and better physical health (Cohen and Wills, 1985; Cohen, 2004; Haber et al., 2007).

It is important to note that the relationship between social support and emotion regulation can be bidirectional. For example, use of expressive suppression during social interactions is associated with lower social satisfaction, lower closeness with others, increased distraction, decreased responsiveness, and impaired memory for conversation content (Marroquín, 2011). Those who habitually use suppression also report feelings of inauthenticity due to an imbalance between inner experience and outer expression, which contributes to avoidant, diverted, and anxious relational behaviors (Gross and John, 2003; John and Gross, 2004; Cutuli, 2014). Consequently, those who interact with someone actively utilizing expressive suppression report higher levels of stress, lower feelings of closeness, and diminished positive feelings toward that person (Gross and John, 2003). Those who habitually use cognitive reappraisal show opposite effects, reporting higher social satisfaction, comparatively closer relationships, higher willingness to share emotions, and were rated as more likable by others (Gross and John, 2003; Cutuli, 2014).

While deficits in emotion regulation have been promoted as targets of focused research and even clinical intervention across multiple disorders, like depression, it is increasingly clear that emotion regulation and social support need to be considered dynamically and may, in fact, be inexorably linked. Thus, we need to further unpack the complex relationships between emotion regulation, social support, and mental well-being (Berking et al., 2014). The goal of our current study was to further investigate, in a large cohort of young adult university students, associations between emotion regulation (both adaptive and maladaptive strategies), PSS, and the experience of negative mood, specifically symptoms of depression and anxiety. Based on the literature reviewed above, we hypothesized that PSS would mediate, at least in part, the relationship between emotion regulation and future negative emotion wherein more frequent use of cognitive reappraisal but not expressive suppression would be associated with lower future symptoms of depression and anxiety through higher perceptions of social support.

## Materials and Methods

### Participants

Data were available from 544 university students (337 women, age range = 18–22 years old, mean age = 19.64) who successfully participated in the Duke Neurogenetics Study (DNS) between September 30th, 2014 and November 21st, 2016, and had completed specific self-report questionnaires at baseline (T1) and within 3 years of baseline participation (T2). The DNS was reviewed and approved by the Duke University Medical Center Institutional Review Board guidelines, and per their guidelines, all participants provided informed, written consent. To be eligible for participation in the DNS, participants were required to be free of the following conditions: (1) medical diagnoses of cancer, stroke, head injury with loss of consciousness, untreated migraine headaches, diabetes requiring insulin treatment, chronic kidney, or liver disease; (2) use of psychotropic, glucocorticoid, or hypolipidemic medication; and (3) conditions affecting cerebral blood flow and metabolism (e.g., hypertension).

As the DNS seeks to examine the broad distribution of dimensional behavioral and biological variables, any past or current DSM-IV Axis I disorder or select Axis II disorders (antisocial personality disorder and borderline personality disorder) was not an exclusion to participation. However, no individuals, regardless of diagnosis, were taking any psychoactive medication during or at least 14 days prior to their participation. Categorical diagnosis was assessed with the electronic Mini International Neuropsychiatric Interview (Lecrubier et al., 1997) and Structured Clinical Interview for the DSM-IV subtests (First et al., 1996). Of the 544 participants included in our analyses, 32 met criteria for major depressive disorder, 12 for bipolar disorder, 13 for panic disorder, 12 for generalized anxiety disorder, 1 for post-traumatic stress disorder, 46 for alcohol abuse, 10 for substance abuse, 4 for eating disorder (bulimia or anorexia), 2 for psychotic symptoms, 9 for obsessive compulsive disorder, and 7 for social anxiety disorder.

### Self-Report Questionnaires

The Mood and Anxiety Questionnaire (MASQ) Short Form, a 62-item self-report questionnaire, was used to assess anxious and depressive symptoms over the past week at baseline and T2. Symptoms of depression and anxiety are scored along four subscales: two General Distress factors (22 items, 11 assessing symptoms relative to depression and 12 assessing symptoms relative to anxiety), an Anxious Arousal factor (17 items), which is specific to anxious symptoms, and an Anhedonic Depression factor (22 items) specific to depression. Items are rated on a 5-point scale ranging from 1 (not at all) to 5 (extremely). (Clark and Watson, 1991). A total MASQ score is generated by summing all items across the four subscales, which have high internal consistency across multiple samples (Watson et al., 1995). To comply with IRB requirements, one item on the Anhedonic Depression subscale relating to suicidality was removed.

The Emotion Regulation Questionnaire (ERQ), a 10-item self-report questionnaire, was used to measure individual differences in Cognitive Reappraisal (6 items) and Expressive Suppression (4 items) at baseline (Gross and John, 2003). All items are rated on a 7-point scale from 1 (strongly disagree) to 7 (strongly agree) and summed within strategy to generate overall scores for reappraisal and suppression.

The Interpersonal Support Evaluation List (ISEL), a 12-item self-report questionnaire, was used to measure an individual’s perception of social support at baseline (Cohen et al., 1985). Items are rated on a 4-point scale ranging from 0 (Definitely False) to 3 (Definitely True).

### Follow-Up Assessments

Participants were re-contacted by email to complete follow-up assessments online every 3 months after baseline and were entered into a raffle for one $50 Amazon gift card for each round of follow-up assessments. During these assessments, which were facilitated through secure Qualtrics links, the participants were asked to complete an additional MASQ among other measures. The same questions from the MASQ were used to assess current depressive and anxiety symptoms during these follow-up assessments. We selected the most recent MASQ available for all participants within 3 years since enrollment to use as our future measure of depression and anxiety.

### Data Analysis

Our hypothesis was that the habitual use of cognitive reappraisal (but not habitual use of suppression) would be associated with lower future symptoms of depression and anxiety, in part, through higher PSS. Path analyses using the programming language R (v 3.4.4.) were performed between ERQ Cognitive Reappraisal subscale and ISEL (Path A), ISEL and future (T2) MASQ (Path B) and ERQ Cognitive Reappraisal and MASQ (Path C) to check for potential mediation (Baron and Kenny, 1986; Zhao et al., 2010). Identical analyses were performed using ERQ Suppression subscale. Baseline MASQ scores, age, sex, and any diagnoses were included as additional covariates to control for potential confounds within the model. All betas were standardized to allow for comparison.

Sample size was determined prior to running any analyses and included all participants within the DNS who’d completed a follow up survey within 3 years of initial participation. We ran a *post-hoc* sensitivity power analysis for multiple regression, which showed our analyses had 90% power to detect an effect size of *R*^2^ = 0.03 at alpha = 0.05, with 5 predictors and 544 subjects. No analyses were run using a different sample size. In this study, we report all measures, manipulations and exclusions.

## Results

### Behavioral Measures

Means and standard deviations for relevant variables were as follows: MASQ total score at baseline (T1) (112.65 ± 27.47, range = 147); MASQ total score at time two (T2) (112.53 ± 26.12, range = 179); ERQ Reappraisal (5.21 ± 0.92, range = 5.5); ERQ Suppression (3.79 ± 1.23, range = 6); ISEL (28.05 ± 5.77, range = 31). MASQ at baseline (T1) significantly predicted future MASQ scores (T2) [β = 0.52, *p* < 0.001, 95% CI (0.46, 0.62)], and accounted for 28% of the variance [*F*(539) = 53.05, *R*^2^ = 0.28, *p* < 0.001].

*Post hoc* analyses revealed no significant sex differences for MASQ(T1) or MASQ(T2). However, average ERQ reappraisal subscale scores were significantly lower [*t*(379.57) = 2.88, *p* = 0.004, 95% CI (0.08, 0.41)] for men (*M* = 5.06, *SD* = 1.02) compared with women (*M* = 5.30, *SD* = 0.85). Average ERQ Suppression subscale scores also differed significantly [*t*(459.8) = -4.40, *p* < 0.001, 95% CI (-0.67, -0.26)], with women (*M* = 3.61, *SD* = 1.24) scoring lower than men (*M* = 4.07, *SD* = 1.16). There were significant sex differences between men and women in the ISEL scale as well [*t*(399.28) = 3.82, *p* < 0.001, 95% CI (0.95, 2.99)], with women (*M* = 28.80, *SD* = 5.44) scoring higher than men (*M* = 26.83, *SD* = 6.08). *Post hoc* analyses showed no significant effect of time between assessments on the model results.

### Indirect Effects Models (Figure 1)

**FIGURE 1:**
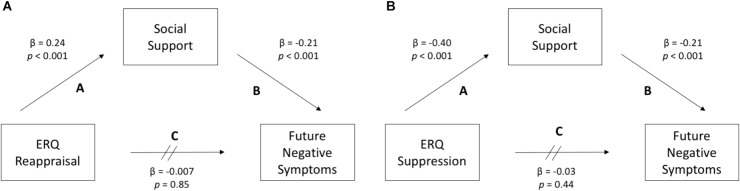
**(A)** The habitual use of cognitive reappraisal does not significantly predict future symptoms of depression and anxiety (Path C). Instead, it is significantly associated with perceived social support (Path A), which in turn significantly predicts future symptoms (Path B). **(B)** The habitual use of expressive suppression also does not predict future negative symptoms (Path C), but is negatively associated with perceived social support (Path A), which in turn predicts future symptoms (Path B).

The habitual use of cognitive reappraisal did not significantly predict future MASQ scores (T2) [β = -0.007, *p =* 0.85, 95% CI (-2.41, 1.99)] above and beyond baseline MASQ scores [Δ*R*^2^ < 0.001, *F*(538) = 0.04, *p* = 0.85]. However, reappraisal was significantly positively correlated with ISEL scores [β = 0.24, *p* < 0.001, 95% CI (0.99, 2.01)], accounting for approximately 10% of the variance [*R*^2^ (539) = 0.09, *p* < 0.001]. In turn, higher ISEL scores significantly predicted lower future MASQ scores (T2) [β = -0.21, *p* < 0.001, 95% CI (-1.38, -0.59)] above and beyond baseline MASQ scores [Δ*R*^2^ = 0.03, *F*(538) = 23.92, *p* < 0.001].

The habitual use of expressive suppression also did not significantly predict future MASQ scores (T2) [β = 0.03, *p* = 0.44, 5% CI (-1.02, 2.37)] above and beyond baseline MASQ scores [Δ*R*^2^ = < 0.001, *F*(538) = 0.61, *p* = 0.44]. However, suppression was significantly negatively correlated with ISEL scores [β = -0.40, *p* < 0.001, 95% CI (-2.24, -1.52)], accounting for approximately 20% of the variance [*R*^2^ (538) = 0.19, *p* < 0.001]. Higher ISEL scores, in turn, significantly predicted lower future MASQ scores, as was true with reappraisal.

*Post hoc* analyses revealed that the patterns observed herein held true for both the depression and anxiety subscales of the MASQ.

## Discussion

Here we found evidence that the association between habitual strategies for regulating negative emotions and the future experience of negative mood is, in part, mediated by PSS. Significant correlations were observed between habitual use of cognitive reappraisal and PSS as well as between PSS and future symptoms of anxiety and depression. There was, however, no direct association between reappraisal and future negative symptoms. This pattern was also observed for the habitual use of suppression, which was associated with PSS, but not directly with future negative symptoms.

These findings build on prior research demonstrating the close link between social support and emotion regulation (Lopes et al., 2005; Marroquín, 2011; Zaki and Williams, 2013; Marroquín and Nolen-Hoeksema, 2015), as well as between social support and future negative symptoms (Cohen and Wills, 1985; Cohen, 2004; Williams and Galliher, 2006). Moreover, our results go further in suggesting that PSS might be a mechanism through which emotion regulation shapes future symptoms of depression and anxiety. One interpretation of our results is that those who commonly use cognitive reappraisal may have higher PSS, which in turn buffer them from future negative symptoms. In contrast, such buffering may not be present for those who commonly use suppression, which is associated with lower PSS (Gross and John, 2003; Cutuli, 2014). Here, it is important to consider that ERQ (John and Gross, 2004) and, to a lesser extent, ISEL (Cacioppo and Hawkley, 2009) capture relatively stable, trait-like features of individuals. Thus, while we assessed these features only at baseline, they likely continue to shape the experiences of participants through the follow-up assessment of mood and anxiety. Consistent with this framework and the results of our primary analyses of longitudinal data, cross-sectional analyses using baseline MASQ revealed that higher reappraisal but not suppression was associated with lower contemporaneous mood and anxiety as a function of higher PSS.

It is important to note the limitations of this study. Foremost, all measures used in the analyses were subjective and collected via self-report. While self-report questionnaires are a common investigative tool in psychology, research suggests that people can lack insight into their cognitive and emotional states or experiences, and thus provide information that may not accurately reflect the underlying processes of interest (Haeffel and Howard, 2010). In addition, the choice of strategies captured by the ERQ are limited in scope. It is possible that there are other habitual emotion regulation strategies commonly utilized by participants that are were not taken into consideration. Future research could employ strategies such as ecological momentary assessment to address these issues. Moreover, the ERQ assesses spontaneous or natural use of cognitive reappraisal and suppression in daily life (Gross and John, 2003). Thus, while the results observed here suggest that the effectiveness of habitual emotion regulation strategies may depend, in part, on social support, it is possible that targeted emotion regulation strategies (e.g., within a clinical context) diverge from this pattern. In addition, the ISEL subscale measures PSS, which is only one aspect of social connectedness (Cohen and Wills, 1985). It is possible that other measures of social support would show alternative patterns of association. Finally, our sample was made up of high-functioning university students, thus future studies in population representative samples are needed to determine the extent to which these links may generalize.

The study of emotion regulation has important clinical implications, and deeper understanding of mechanisms through which these strategies impact risk for psychopathology is needed for continued progress. Our findings suggest that future studies looking to further delineate the relationships between emotion regulation and the experience of depression and anxiety could benefit from explicit assessment of social support networks. If these results are replicated in clinical settings, they would highlight the potential of social support networks as adjunct targets in intervention strategies, such as cognitive behavioral therapy, involving increased use of cognitive reappraisal (Sloan et al., 2017).

## Data Availability

The raw data from the Duke Neurogenetics Study supporting the conclusions of this manuscript will be made available by the authors to any qualified researcher upon request.

## Author Contributions

Td’A developed the study concept. Data analyses were performed by Td’A and KF under the supervision of AH. BB designed the clinical interview and neuropsychological testing protocols. SR and AK collected the data. Td’A, KF, and AH drafted the manuscript. AH conceived and designed the parent Duke Neurogenetics Study. All authors reviewed and approved the final version of the manuscript.

## Conflict of Interest Statement

The authors declare that the research was conducted in the absence of any commercial or financial relationships that could be construed as a potential conflict of interest.
